# Fun During Knee Rehabilitation: Feasibility and Acceptability Testing of a New Android-Based Training Device

**DOI:** 10.2174/1874431101711010029

**Published:** 2017-08-10

**Authors:** Thomas Sanjay Weber-Spickschen, Christian Colcuc, Alexander Hanke, Jan-Dierk Clausen, Paul Abraham James, Hauke Horstmann

**Affiliations:** Institute of Sports Medicine and Trauma Department, Hannover Medical School, Carl-Neuberg-Str. 1, 30625 Hannover, Germany

**Keywords:** Android-based, Computer game, Fast track, Knee, Rehabilitation, Arthroplasty, CAMOped, ARTROMOT^®^ ACTIVE-K

## Abstract

**Purpose:**

The initial goals of rehabilitation after knee injuries and operations are to achieve full knee extension and to activate quadriceps muscle. In addition to regular physiotherapy, an android-based knee training device is designed to help patients achieve these goals and improve compliance in the early rehabilitation period. This knee training device combines fun in a computer game with muscular training or rehabilitation. Our aim was to test the feasibility and acceptability of this new device.

**Methods:**

50 volunteered subjects enrolled to test out the computer game aided device. The first game was the high-striker game, which recorded maximum knee extension power. The second game involved controlling quadriceps muscular power to simulate flying an aeroplane in order to record accuracy of muscle activation. The subjects evaluated this game by completing a simple questionnaire.

**Results:**

No technical problem was encountered during the usage of this device. No subjects complained of any discomfort after using this device. Measurements including maximum knee extension power, knee muscle activation and control were recorded successfully. Subjects rated their experience with the device as either excellent or very good and agreed that the device can motivate and monitor the progress of knee rehabilitation training.

**Conclusion:**

To the best of our knowledge, this is the first android-based tool available to fast track knee rehabilitation training. All subjects gave very positive feedback to this computer game aided knee device.

## 1 INTRODUCTION

The 21^st^ century is characterized by the effort to implement new technologies into all parts of our life. The use of computer game assisted rehabilitation was found to be effective in patients who suffer from stroke and other neurological disorders like Parkinson’s disease and multiple sclerosis [1-3]. Commercial video games such as the development of Nintendo Wii^®^ and its software ‘Wii Fit balance board’ proved their effectiveness in upper limb mobility rehabilitation and lower limb balance control training [4, 5]. Computer game assisted rehabilitation was also recommended after ligament reconstruction knee surgeries to improve knee joint mobility and proprioception [6, 7]. However, currently this rehabilitation tool usually comes up in the later stage of rehabilitation training.

Knee surgeries are one of the most common surgeries in the field of orthopaedics and trauma surgery [6]. Especially in case of competitive sports, knee injuries often result in long downtimes and force top athletes to a premature termination of the career. It is well known that the surgical outcomes are in large part determined by the postoperative treatment. This means that apart from patients receiving proper physio and occupational therapy, there is also a great need for patients to do exercises independently in accordance with the instructions received from physiotherapists. Therefore, basic movements and range of motion need to be taught and patient-performed exercises which are similar to exercise programs done in training, need to be supported. However, in most cases, the transfer of training techniques by the physician into successful and desired postoperative goals is not a simple task. A training technique needs to be developed which is self-explanatory, effective, encouraging and accessible to the patient all at the same time. Such a technique, in comparison to competitive sports, would also have to be interesting and motivating for the patient in enhancing the desired postoperative treatment after a standard surgical intervention such as knee arthroplasty, proximal tibial osteotomy or supracondylar osteotomy.

Arthrogenic muscle inhibition is an ongoing neural activation deficit of the quadriceps muscle group after knee injury, knee joint arthritis or knee surgery [1]. Therefore, it is very important to achieve full knee extension and quadriceps muscle group activation at the initial stage of knee rehabilitation [4]. However, most patients are reluctant or perhaps not motivated enough to start their early knee rehabilitation for several reasons. These factors could be attributed to persistent pain and swelling of the knee, bulky wound-dressings, the presence of operative drains that limit the knee motion, or lower limb weakness after spinal anesthesia and peripheral nerve block [2, 8]. Physiotherapists can sometimes have difficulties building up a good and sustainable working rapport with the patients as a result of postoperative pain and uncertainty on given limits on the side of the patient at the start of the rehabilitation program [9].

We developed an android-based knee-training device to interactively support patients to achieve full knee extension and avoid quadriceps muscle group atrophy immediately after knee injury or surgery [10]. With the aid of the App-based knee-trainer, patients can be motivated to start their rehabilitation even before they receive elective knee surgery and are encouraged to perform these exercises independently during their hospital stay in addition to regular physiotherapy [11]. Utilizing the App-based knee-trainer, the compliance and the progress of rehabilitation can be monitored by the patients as well as the surgeons or the physiotherapists [12].

Through this device, the compliance and the progress of rehabilitation can be monitored by both patients and their doctors or physiotherapists. It aims to provide a fast track recovery that enables a shorter hospital stay and therefore reduces social as well as hospital costs.

The aim of this pilot study was to test the feasibility of this device and the users’ acceptability and response towards this new tool.

## 2 MATERIALS AND METHODS

### 2.1 The Knee Device

**Fig. (1):**
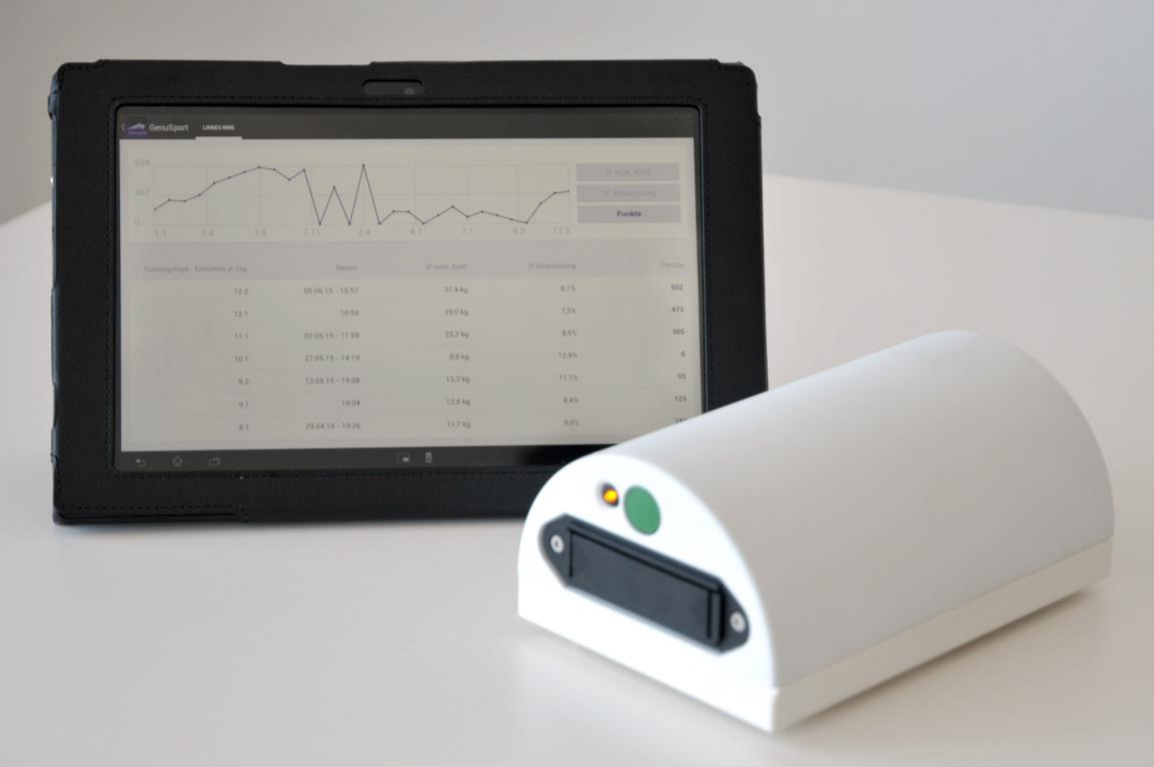
Knee training device with an android-based tablet.

This device was originally invented and produced, aiming to help patients to resume full knee extension and activate quadriceps muscle *via* an android-based computer game. The prototype, developed by the senior knee surgeon Dr. Weber-Spickschen and constructed by the Department of Medical Device Construction of the Hannover Medical School, Germany. It measures 18cm (length) x 12cm (width) x 8cm (height) in size and runs on 4AA batteries. It is composed of 3 Arduino sensors, 2 LED lights and an electric circuit board. It can be connected to any ordinary android-based tablets using Bluetooth function (Fig. **1**).

### 2.2 The Computer Game

The users were instructed to place the knee device underneath their popliteal fossa and connect the device to their android-based tablet computer using the Bluetooth function as shown in Fig. (**2**). The game was divided into 2 parts. The language used for instruction was German.

**Fig. (3):**
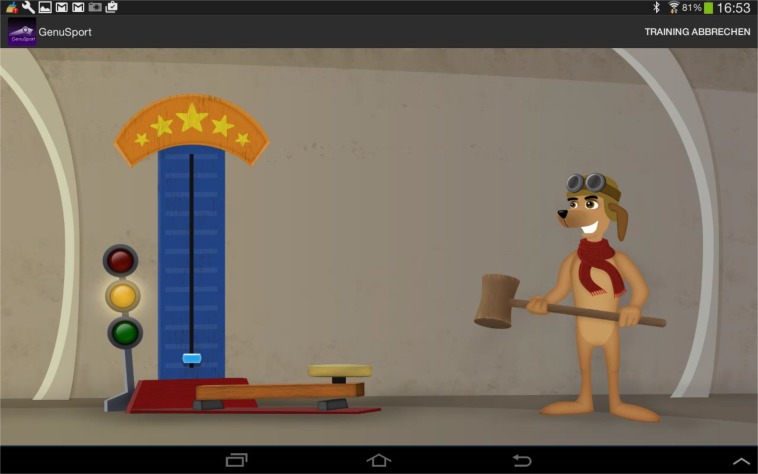
High striker game – illustration on the tablet of the maximum reachable power of knee extension.

The first game was the high striker game (Fig. **3**). Subjects were instructed to extend their knee and press their popliteal fossa as hard as they can on the knee device for 5 seconds, to achieve a maximum score. The maximum power of knee extension was measured and recorded.

**Fig. (4):**
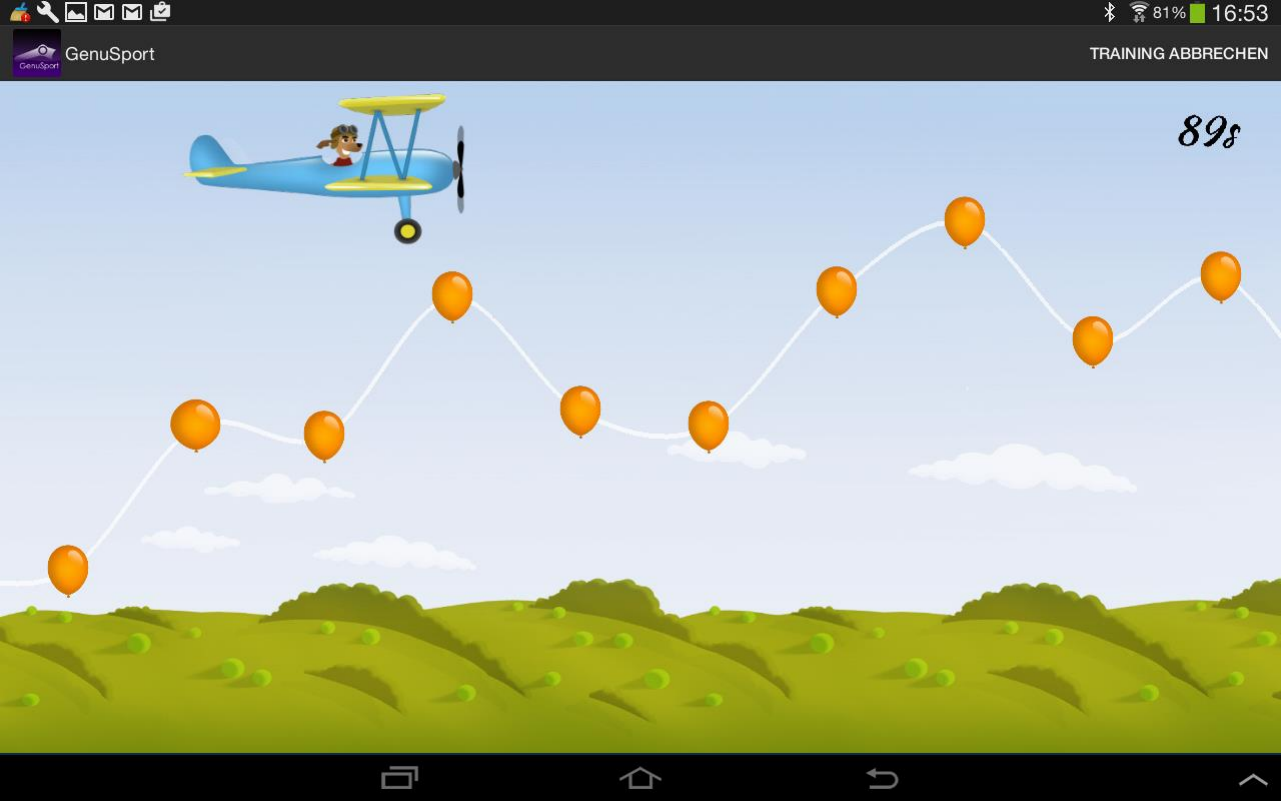
Aeroplane flying simulation curve representing the maintenance of knee extension und accuracy of quadriceps muscle control. The generated path should be taken and the balloons should be hit to achieve maximum score.

The second game was a simulation of piloting a plane for duration of 100 seconds. This was achieved by extending the knee and pressing the popliteal fossa against the knee device. The stronger the force, the higher the aeroplane will fly. In order to achieve a higher score, the plane needed to follow a designated curve and hit the balloons along the path with the front propeller as it flies (Fig. **4**). The curve was generated using the maximum power achieved from the first game and previous training data. A more difficult curve will be generated if a higher score was achieved in the previous session. Furthermore, scores will be deducted if the plane flies into the dark clouds. The frequency of dark clouds occurrence is determined by previous past training records.

**Fig. (5):**
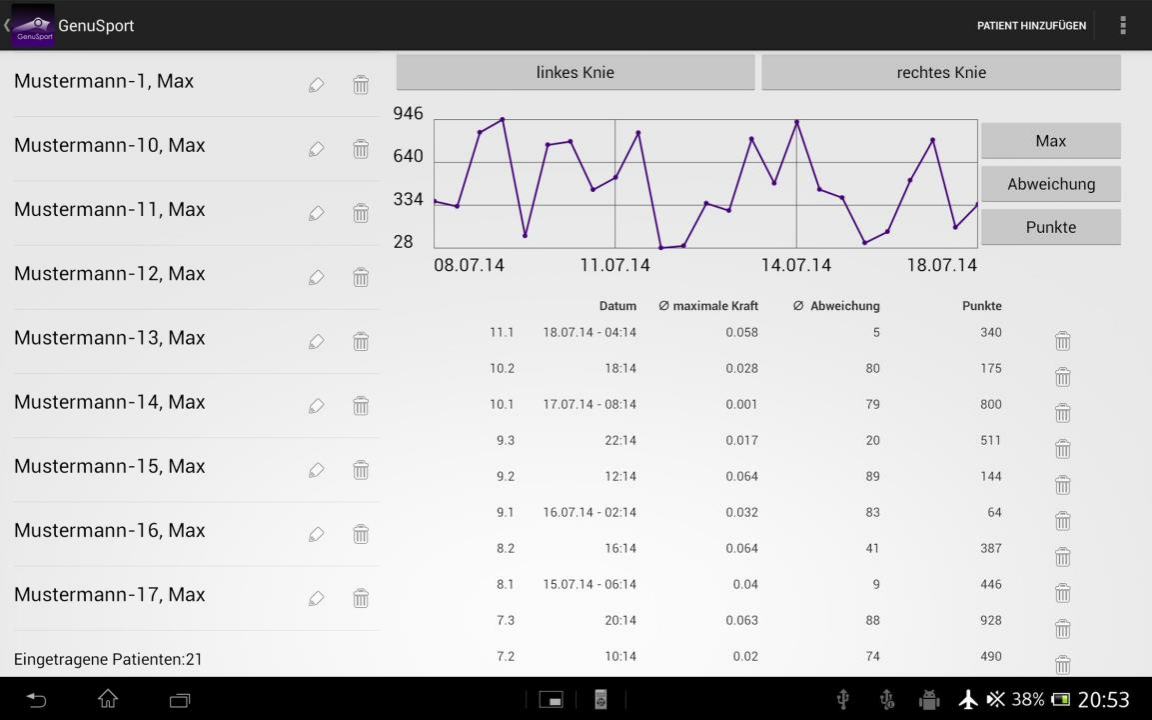
The android app automatically generates and saves the details of the training session. These details can be readily accessible for making comparisons and following the progress.

The training time and frequency of training, the maximum knee extension power, the accuracy of quadriceps muscle activation and the final scores were all recorded in the game (Fig. **5**). This logbook was kept as a record for both patients and their physiotherapists and doctors for further clinical evaluation.

### 2.3 The Pilot Study

50 healthy volunteers (37 male and 13 female) aged 18 - 40 (mean 21.7) were recruited from the Olympic Centre, Lower Saxony Hannover, Germany to test out this new knee device. The participation was voluntary. Nine (18%) subjects sustained previous knee injury, including 2 anterior cruciate ligaments (ACL) injuries and 2 meniscus injuries. None of the subjects presented acute knee symptoms before using this knee device. Subjects’ demographic data is shown in Table **1**. Subjects responded to a simple questionnaire in German language to assess the use of the device, followed by an evaluation of the doctor in charge. There was a possible technical issue in the main parameter. Second-line parameter was the subjects’ satisfaction rated in the questionnaire.

## 3 RESULTS

All 50 subjects completed a single session of using this knee rehabilitation device for both the knees. The drop-out rate was zero and all subjects completed the questionnaire. There was no computer-related technical problem identified. All users rated either ‘excellent’ or ‘very good’ overall experience with this product. No subjects complained of any discomfort or worsening of knee symptoms during or after using this device. In the questionnaire, all users were convinced that this product will help to motivate patient and improve the compliance during early knee rehabilitation.

In the high striker game, the mean maximum knee extension power recorded was 22.6 kg for dominant and 21.7 kg for non dominant limbs. In the second aeroplane flying simulation game where knee muscle activation and control were evaluated, the mean deviation from designated curve was 7.5% for dominant and 7.3% for non-dominant limbs. The average total high score for both the combined games was 439.9 for dominant and 422.9 for non-dominant leg (Table **2**).

## 4 DISCUSSION

The results of the pilot study are encouraging. No technical problem was identified in this study. In the subjective results of the questionnaire to the overall experience, the ratings were “excellent” or “very good”.

The app-based knee-trainer is to the best of our knowledge one of the only medical devices that offer the advantages of a location-independent, easily and autonomously feasible exercise training, combining strength and motion, which is translated in a computer game, that enhances fun driven postoperative rehabilitation.

Quadriceps strength and endurance are of vital importance for normal knee function and full knee extension is also needed in a normal gait cycle [13]. Several researches have been conducted in the past to promote knee activation and improve muscle strength to reduce post-traumatic quadriceps weakness. Both early initiation of progressive eccentric and concentric exercise [14] and whole body vibration therapy [15] showed improvement in functional outcome and muscle performance after ACL reconstruction. The combined use of eccentric exercise and neuromuscular electrical stimulation also helped to restore quadriceps activation and strength after total knee arthroplasty [16]. A recent article recommended the use of continuous passive motion machine with vibration function to prevent flexion contracture in knees [17]. In the era of technology, robot assisted rehabilitation training showed improvement in joint proprioception and stability after knee arthroplasty [18].

Since compliance to physiotherapy is often poor in the first few days after knee trauma, the authors wish to motivate patients during early knee rehabilitation through a computer game aided rehabilitation that is integrated with fun and physical training. Patients can start rehabilitation as soon as they wake up from anaesthesia or even before they undergo elective operations. This aims to facilitate quadriceps reactivation and prevent post-traumatic knee weakness. It is believed that by playing computer game, post-traumatic pain can be reduced by mental distraction, similar to the effect of listening to music that can reduce pain after knee arthroplasty [19]. Nevertheless, further study is needed to confirm this statement.

Both the high striker game and the aeroplane simulation flying game aim to achieve full knee extension and precise muscle activation. A precise control of the knee power is needed to fly the plane at the aimed flying curve. Accuracy of muscle firing is calculated by the deviation between the designated flying curve and the actual flying curve. These data help to monitor the users’ rehabilitation progress and check the compliance. With the help of this new device, authors believe that users should feel more responsible and more motivated for their own rehabilitation training. Since the data can be assessed by doctors and physiotherapists at the clinic, it serves as an excellent platform for physiotherapists and doctors to monitor patients’ progress, even when patients live far away from hospital. The duration and frequency of rehabilitation training can be maximised as the exercise can be performed alone at anytime without supervision. Using the training data, physiotherapists and doctors can also design the most suitable rehabilitation protocol for patients. This new knee device can promote fast track knee rehabilitation and facilitate ambulatory day surgery.

A limitation of this pilot study is its small sample size. Most of the subjects recruited in this study are young elite athletes, a subject group with best coordination and which can easily adapt to computer game aided rehabilitation. In future clinical study, it is important that similar response be achieved in patients who are symptomatic and older and not familiar with playing computer games. Furthermore, validity of this study can be improved by multiple training sessions for an extended period of time.

In the recent literature, there is only one product known that can be described as a computer game-based rehabilitation knee-device named the LegTutor. This is an orthosis that provides a functional and fine motor rehabilitation of the leg, imaging the joint's motion on a computer screen. In contrast to this device, the app-based knee-trainer is able to measure the force generated during knee extension training and display the validated values wirelessly in real-time as a computer game.

There are two training devices which can be used for active training in the early postoperative rehabilitation. Both devices [CAMOped and the ARTROMOT^®^ ACTIVE-K) do have the opportunity to set the range of motion and the resistance to muscle power. In comparison to the knee-trainer, they have no option of collecting and storing data for the therapists. In addition, the knee-trainer has the advantage of training with a computer application-based training game.

Furthermore, there are knee-training devices which can be used for continuous passive motion training. These devices lack the ability of active muscle training and have less potential of proprioceptive improvement.

Besides the above mentioned knee training devices, there are many apps for knee pain available (*e.g*. “Knee Pain Relieving Exercises by Dr. Kavin Khatri”, “Knees Therapie by Expert Health Studio” or “knees therapy by Health Care Tips”). In contrast to our android-based training device, the common aspect in these apps is that they do not have a real-time force measurement. On account of this, our android-based training device has the advantage of giving the user a biofeedback while playing the aeroplane game.

Future study will need to investigate which muscles around the knee are activated when the popliteal fossa is pressed against the knee sensor. Further studies would evaluate whether maximum power can be improved by playing the game more frequently. It will be interesting to find out whether functional recovery after knee arthroplasty or ligament reconstruction surgeries can be improved by adding this device into the existing postoperative rehabilitation protocol. With increasing recognition and acceptance of the device, the authors hope that specific computer game programs could be designed for different knee operations and in different age groups. An English version of the device can reach more patients on a global level.

## CONCLUSION

The initial goals of rehabilitation after knee injuries and operations are to achieve full knee extension and to bring the quadriceps muscle group back to pre-injury strength. In addition to regular physiotherapy, a special knee device with an android-based application visualizing the training as a computer game is designed to assist patients to achieve these goals and improve compliance in the early rehabilitation.

The users’ feedbacks on this device are encouraging. No technical problem was identified in this study. Future clinical studies will be conducted to test its effectiveness in real patients.

## Figures and Tables

**Table 1:** Demographic data.

Age (y), mean ± SD	21.7 ± 4.6
Sex (Male / Female)	37/13
Body weight (kg), mean ± SD	77.4 ± 12.4
Body height (m), mean ± SD	1.81 ± 0.08
BMI (kg/m^2^), mean ± SD	23.4 ± 2.7
Previous knee injury	9

**Table 2:** Performance of subjects in the “high striker” and “Aeroplane flying” game.

High Striker Game (in kg)		Flying Game (in %)		Total High Score	
Dominant leg	Non dominant leg	Dominant leg	Non dominant leg	Dominant leg	Non dominant leg
22.6 ± 8.9	21.7 ± 7.7	7.5 ± 4.0	7.3 ± 2.3	439.9 ± 170.5	422.9 ± 142.4

**Fig. (2):**
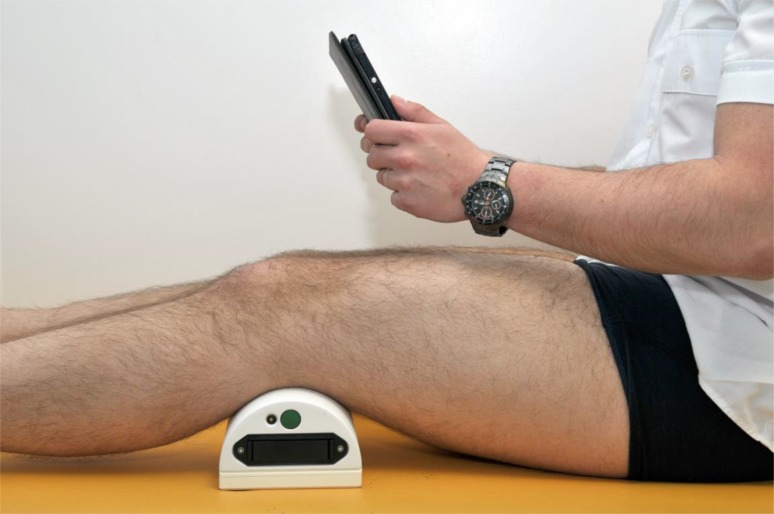
Start position with training device directly underneath the subject’s knee. The tablet is wirelessly connected to the device through a Bluetooth function.
